# Supplementary material for: Unconventional magnetism mediated by spin-phonon-photon coupling
Source: Nat Commun. 2024 May 11;15:4000. doi: 10.1038/s41467-024-48404-z (PMC11088681; doi:10.1038/s41467-024-48404-z)
Supplement: Supplementary file 1 — Supplementary Information [file 41467_2024_48404_MOESM1_ESM.pdf]

Supplementary Information: Unconventional magnetism mediated by spin-phonon-photon coupling

Petros Andreas Pantazopoulos, Johannes Feist, Francisco J. García-Vidal, and Akashdeep Kamra

Departamento de Física Teórica de la Materia Condensada and Condensed Matter Physics Center (IFIMAC), Universidad Autónoma de Madrid, Madrid, E-28049, Spain.

Corresponding author(s). E-mail(s): petros.pantazopoulos@uam.es; johannes.feist@uam.es; fj.garcia@uam.es; akashdeep.kamra@uam.es;

1 Supplementary Note 1: Spin-phonon coupling

In this note, we derive the spin-phonon coupling term employed in the main text within a simplified and general framework. This also offers guidance with respect to the materials relevant for our proposal. We first motivate the spin-phonon coupling term relevant to our analysis on symmetry grounds. Then, we briefly review the well-established theory of spin-phonon or magnetoelastic coupling for ferromagnets and acoustic phonons [1–3]. This analysis is then generalized to the case of lattice with a basis thereby formulating spin-phonon coupling for acoustic and optical phonons in a unified framework. This allows us to establish additional symmetry requirements on the materials that may host our considered spin-phonon coupling term. In addition, the developed framework enables an estimation of the spin-optical phonon coupling based on the more widely available measurements of magnetoelastic constants in magnets [2].

1.1 General form on time-reversal symmetry grounds

The term "spin-phonon coupling" has been employed to discuss a broad range of distinct effects in the literature. Hence, we must first clarify the particular term that we are interested in and justify its relevance. To this end, we begin by considering the lowest order terms in the combined potential energy density for the spin and phonon system. This approach forms the foundation of conventional magnetoelasticity theory [1]. The lowest order terms are

1. $\sim \widehat{S}_k Q$
2. $\sim \widehat{S}_k Q^2$
3. $\sim \widehat{S}_k^2 Q$
4. $\sim \widehat{S}_k^2 Q^2$
5. $\sim \nabla \widehat{S}_k Q$
6. $\sim \nabla \widehat{S}_k Q^2$
7. $\sim \left(\nabla \widehat{S}_k\right)^2 Q$
8. $\sim \left(\nabla \widehat{S}_k\right)^2 Q^2$

where $\widehat{S}_k \equiv \widehat{S}_k(\mathbf{r}) = S_k(\mathbf{r})/S$ represents the direction cosine of the position dependent spin profile with k representing a Cartesian coordinate, and Q is the generalized displacement coordinate representing the phonon mode in question. In the above list, terms 1, 2, 5, and 6 are forbidden by time-reversal symmetry requiring the Hamiltonian to remain the same under the substitution $\widehat{S}_k \rightarrow -\widehat{S}_k$. The terms 5 through 8 can further be considered higher-order since they involve the gradient of the spin direction cosine in a ferromagnetic ground state and are also disregarded, similar to what is done in the conventional magnetoelasticity theory [1]. This leaves terms 3 and 4, with the 4th being higher order than the third. Hence, in this work, we focus on the term 3, $\sim \widehat{S}_k^2 Q$, the effect of which on the ground state of a spin system has not been considered before. On the other hand, and in contrast with term 3, the term 4 gives rise to a shift in the phonon frequency depending on the magnetic state and has been more widely investigated.

Therefore, the time-reversal symmetry alone warrants that in a strong ferromagnet the leading order effect of spin-phonon coupling is captured by a term $\sim \widehat{S}_k^2 Q$. However, since we focus on a very specific phonon mode in this work - the zero wavenumber infrared (IR) active optical mode - additional symmetry constraints due to the crystal structure may be present. We examine this in more detail below.

1.2 Magnetoelasticity theory and spin-pair model

Besides relying on time-reversal and crystal symmetry arguments, the conventional magnetoelasticity theory can be constructed using Néel's spin-pair model [2, 4] depicted in Fig. S1. It considers a pair of aligned spins making an angle θ with the position vector that separates the spins by a distance R.

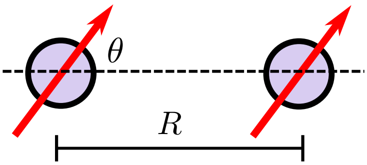

Fig. S1 A pair of aligned spins make an angle θ with the position vector separating them by a distance R.

Within a simplified model, it is assumed that the spin-pair interaction energy W depends on R and θ, thereby admitting a Taylor expansion in terms of even spherical harmonics [2]:

$$W(R,\theta) = g(R) + l(R)\left(\cos^2\theta - \frac{1}{3}\right) + \cdots, \tag{S1}$$

where $g(R)$ and $l(R)$ parametrize the separation dependence of the various terms, and we only show the first two even spherical harmonics. The odd ones are forbidden by, again, invariance under time-reversal. Here, the term $g(R)$ independent of θ includes contribution from exchange interaction and does not depend on the spin direction. Thus, it does not cause spin-phonon coupling, but instead a renormalization of the elastic forces and equilibrium lattice configuration. We are not interested in this effect here and thus may approximate

$$W(R,\theta) \approx l(R)\cos^2\theta. \tag{S2}$$

The physical origin of this spin-lattice coupling term is material-dependent with contributions from spin-orbit interaction causing single-ion anisotropies, magnetic dipolar fields and so on [1, 2, 5].

Considering how a strain affects the distance R and direction θ associated with the spin-pair, one may evaluate the resulting change in W. Summing over all the spin-pairs in the unit cell, one obtains an expression of the form:

$$H_{\mathrm{mec}} = \int \mathrm{d}\mathbf{r} \sum_{k=x,y,z} b_1 \frac{S_k^2}{S^2} u_{kk} + \int \mathrm{d}\mathbf{r} \sum_{k,k'=x,y,z} b_2 \frac{S_k S_{k'}}{S^2} u_{kk'}, \tag{S3}$$

for the magnetoelastic energy in a cubic crystal [1, 2]. Here, $u_{kk'} \equiv 1/2(\partial R_k/\partial x_{k'} + \partial R_{k'}/\partial x_k)$ are strain tensor components, $b_{1,2}$ parametrize the spin-phonon coupling, and $S_k \equiv S_k(\mathbf{r})$ are the components of spatially resolved spin. In ferromagnetic nanoparticles, the so-called macrospin approximation is valid due to the internal exchange being strong. Thus, the spin components do not depend on the position within the nanoparticle and the integral in the equation above simply yields a volume factor thereby enabling an adequate description in terms of the total nanoparticle spin or its direction (see

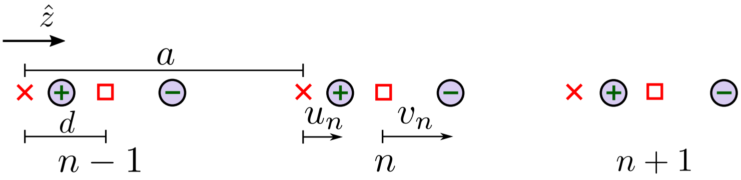

Fig. S2 A diatomic chain model for a two-sublattice magnet hosting acoustic and polar optical phonons [11]. The equilibrium locations of the two kinds of atoms (circles) are marked by red crosses and squares. Here, a is the lattice constant and d is distance between the two atoms in the basis. The atomic displacements from their respective equilibrium positions are described by u_n and v_n.

Sec. 1.6). The parameters $b_{1,2}$ can be evaluated in terms of the function $l(R)$ as shown below, but are typically treated as experimentally determined material parameters.

In considering acoustic phonons, one simply replaces the strain components in the equation (S3) above by the relevant phonon displacements [3]. While the above has been evaluated for cubic crystals and acoustic phonons, the main form and features of the relations go well-beyond. They also work for optical phonons [6, 7] as well as polycrystalline materials [8]. This is because the main physics relates to how the spin-pair energies are affected by rotation and extension/compression, which can be treated on a more generic footing [9]. Due to similar generality reasons, the same expression Eq. (S3) has also been employed, successfully and extensively, for multisublattice ferrimagnets [10], such as yttrium iron garnet, despite their highly complex unit cell.

1.3 Diatomic chain model

In this section, we develop a description of spin-phonon coupling within a simple diatomic chain model [11] that explicitly accounts for two sublattices and optical phonon modes. A key goal is to use the spin-pair model in addressing the spin-phonon coupling for acoustic and optical phonon branches within a unified framework. Our simplified model is meant to establish and guide general symmetry and phenomenological considerations for multisublattice magnets, which are not easily treated analytically.

The model [11], depicted in Fig. S2, consists of a one-dimensional lattice with a basis comprising two oppositely charged atoms displaced by a distance d. As a result, the effective spring constants between the neighboring atoms are different depending on the bond distance being d or $a-d$ resulting in the potential energy:

$$U = \sum_n \frac{1}{2} K \left(u_n - v_n\right)^2 + \frac{1}{2} G \left(u_{n+1} - v_n\right)^2, \tag{S4}$$

where K and G are the two effective spring constants. Assuming the same mass M for the two atoms for simplicity, we obtain the dynamical equations

of motion:

$$M\ddot{u}_n = -\frac{\partial U}{\partial u_n} = -K(u_n - v_n) - G(u_n - v_{n-1}), \tag{S5}$$

$$M\ddot{v}_n = -\frac{\partial U}{\partial v_n} = -K(v_n - u_n) - G(v_n - u_{n+1}). \tag{S6}$$

Considering periodic boundary conditions, we assume plane wave solutions of the form

$$u_n = \mathrm{Re}\left(\tilde{u}e^{i(qna-\omega t)}\right), \tag{S7}$$

and similar for v_n. Substituting these in the equations of motion (S5) and (S6), we obtain the eigenspectrum and eigenmodes:

$$\omega^2(q) = \frac{K+G}{M} \pm \frac{\sqrt{(K-G)^2 + 4KG\cos^2(qa/2)}}{M}, \tag{S8}$$

$$\frac{\tilde{u}}{\tilde{v}} = \mp \frac{K + Ge^{-iqa}}{\sqrt{(K-G)^2 + 4KG\cos^2(qa/2)}}. \tag{S9}$$

The lower (upper) sign in the above expressions corresponds to the acoustic (optical) branch that yields in-phase $\tilde{u} = \tilde{v}$ (out-of-phase $\tilde{u} = -\tilde{v}$) displacements of the atoms for $q = 0$ mode. Furthermore, it can be seen that substituting $K = G$ (corresponding to $d = a/2$) effectively results in a single phonon branch that is continuously connected at the Brillouin zone boundary $q = \pm\pi/a$. This is expected from a single-sublattice system the eigenmodes of which have been written down using our two-sublattice notation and thus a smaller Brillouin zone.

When considering a spatially homogeneous electric field $E\hat{z}$, i.e., within the long-wavelength approximation as applicable for nanoparticles smaller than the relevant wavelengths, the equations of motion (S5) and (S6) are modified as follows

$$M\ddot{u}_n = -K(u_n - v_n) - G(u_n - v_{n-1}) + Q_aE, \tag{S10}$$

$$M\ddot{v}_n = -K(v_n - u_n) - G(v_n - u_{n+1}) - Q_aE, \tag{S11}$$

where Q_a is the charge magnitude for both atoms. This results in the electric field coupling to $u_n - v_n$ and consequently the $q = 0$ optical mode that corresponds to $\tilde{u} = -\tilde{v}$ [Eq. (S9)]. Hence, for coupling to IR light within our polar phonon model, we are primarily interested in the $q = 0$ mode of the optical branch.

Having established the phonon eigenmodes, we turn to evaluating the spin-pair energies assuming, as before, that all the atomic spins are aligned. The

spin energy [Eq. (S2)] involving the two spin-pairs is

$$W_S = \sum_n l_K(R_K)\cos^2\theta_K + l_G(R_G)\cos^2\theta_G, \tag{S12}$$

where the subscripts K and G label the two bonds within a unit cell. The spin-pairs' distances will be affected by longitudinal phonon modes along the z direction while transverse phonons influence $\theta_{K,G}$. Here, we focus on the longitudinal phonon modes and thus, treat $\theta_K = \theta_G = \theta$ as static variables resulting in $\cos\theta$ becoming the third directional cosine of the spin: $\widehat{S}_z$. The spin energy may thus be expressed as:

$$W_S = \sum_n \widehat{S}_z^2 \left(l_K(d) + l_G(a-d)\right) + \widehat{S}_z^2 \left[l'_K(v_n - u_n) + l'_G(u_{n+1} - v_n)\right], \tag{S13}$$

where we have employed $R_K = d + v_n - u_n$ and $R_G = a + u_{n+1} - d - v_n = a - d + u_{n+1} - v_n$ (see Fig. S2), Taylor expanded $l_{K,G}$ functions retaining only the first order terms in the phonon displacements, and employed the concise notation $l'_K(d) \equiv l'_K$ and $l'_G(a-d) \equiv l'_G$. Disregarding the constant offset stemming from the equilibrium configuration, we obtain the spin-phonon coupling energy ΔW_S as:

$$\Delta W_S = \sum_n \widehat{S}_z^2 \left[l'_K(v_n - u_n) + l'_G(u_{n+1} - v_n)\right], \tag{S14}$$

where we need to express the atomic displacements in terms of the phonons.

Let us first consider the acoustic branch and examine phonons with small, but finite, wavenumber q such that $qa \ll 1$. Employing the lower sign in Eq. (S9) and assuming $u_n = u_0\cos(qan)$, we obtain

$$\frac{\tilde{v}}{\tilde{u}} = 1 + \frac{iGqa}{K+G}, \tag{S15}$$

$$v_n - u_n = -\frac{G}{K+G} qau_0 \sin(qan), \tag{S16}$$

$$u_{n+1} - v_n = -\frac{K}{K+G} qau_0 \sin(qan). \tag{S17}$$

Employing these relations in Eq. (S14), we obtain the spin-phonon energy

$$\Delta W_S = \sum_n -\left(l'_K \frac{G}{K+G} + l'_G \frac{K}{K+G}\right) qau_0 \sin(qan)\widehat{S}_z^2, \tag{S18}$$

which is our desired result. Since $qau_0\sin(qan) \sim \partial u(z)/\partial z$ in a continuum model, Eq. (S18) validates Eq. (S3) for longitudinal phonons with wavevector along z direction. It further allows us to obtain a simplified microscopic relation

$b_1 \propto l'_K G/(K+G) + l'_G K/(K+G)$. We can also see now why the simple single-sublattice model that would assume $K = G$ and $l'_K = l'_G \equiv l'$ works well for two(multi)-sublattice systems as it will simply imply $b_1 \propto l'$ without changing the overall coupling form. Assuming $K \sim G$, the estimated value of b_1 is also obtained correctly.

Equipped with this understanding of acoustic phonons, let us examine the optical phonon branch. Since we are interested in the IR active optical mode at $q = 0$, we disregard even the first order terms in q here. Considering the upper sign in Eq. (S9) and assuming $u_n = u_0 \cos(qan)$ as before

$$\frac{\tilde{v}}{\tilde{u}} = -1, \tag{S19}$$

$$v_n - u_n = -2u_0 \cos(qna) = -2u_0, \tag{S20}$$

$$u_{n+1} - v_n = 2u_0 \cos(qna) = 2u_0, \tag{S21}$$

which, employing Eq. (S14), yields

$$\Delta W_S = \sum_n (l'_G - l'_K)\, 2u_0 \widehat{S}_z^2, \tag{S22}$$

for the spin-phonon coupling. Assuming $|l'_G| \ll |l'_K|$, we notice that the spin-phonon coupling above can be obtained from its acoustic phonon counterpart Eq. (S18) by substituting $q \sim 2/a$. This relation and procedure is advantageous/useful since the parameter b_1 that enters Eqs. (S3) and (S18) is easily obtained from experiments in a large variety of magnets. On the other hand, to the best of our knowledge, the corresponding parameter for the spin-optical phonon coupling has not been investigated in detail.

1.4 Consideration of inversion symmetry

In the previous subsection, we have related the strength of spin-optical phonon coupling to its acoustic counterpart based on the assumption $|l'_G| \ll |l'_K|$ and within the diatomic model introduced in Ref. [11] for studying optical phonons. In reality, we need a weaker condition $|l'_G - l'_K| \sim l'_K$ for this estimate. Such a condition is ensured by the different bond distances d and $a - d$ in our considered model. We notice that this necessarily breaks the inversion symmetry. While in our model the distances d and $a - d$ need to be different for the existence of optical phonon modes, this is not generically true since we could have obtained the second phonon branch by simple assuming different masses for the two atoms. On the other hand, to justify $l'_G \neq l'_K$ between the two same spins, we necessarily need the two spin bonds to be different thereby breaking inversion symmetry. Thus, we conclude that our considered coupling between the spin and $q = 0$ IR active phonon is only available in noncentrosymmetric magnets.

Building on these microscopic insights, we now consider a direct argument to arrive at the same conclusion. Assuming spatial inversion symmetry (due

to the crystal structure), only a phonon mode that is odd under spatial inversion may couple to the spatially homogeneous electric field associated with IR light. This is because such a field is odd under spatial inversion while we need the light-phonon coupling term to be invariant under spatial inversion to respect the crystal symmetry. Such a phonon mode cannot directly couple to our assumed aligned spin since the latter is invariant under spatial inversion. Any such coupling between the odd-under-inversion phonon and even-under-inversion spin will violate the inversion symmetry of the crystal.

1.5 Materials

Thus, our considered spin-IR active phonon coupling term is available in noncentrosymmetric magnets. We can thus consider materials with a crystal structure that violates inversion symmetry. At the same time, centrosymmetric materials in the bulk can still become noncentrosymmetric under a strain-gradient [12] that may be deliberate or accidental. For example, the surface of a ferromagnetic nanoparticle is expected to be highly and nonuniformly strained. Along these lines, buckling of thin layers has also been proposed to convert a centrosymmetric material into a noncentrosymmetric one thereby activating additional couplings [13].

Returning to noncentrosymmetric magnets, a large number of candidates exist and some of them have been studied intensely in recent years due to their hosting skyrmions [14, 15]. For our considered nanoparticles, the magnetic ground state of each will remain uniform and without any skyrmions, in consistence with our assumptions. One prominent material example is Cu_2OSeO_3, in which a phonon magnetochiral effect resulting from Dzyaloshinskii-Moriya interaction has already been observed [16]. However, the particular spin-phonon coupling parameter relevant to our proposal has not been probed, to the best of our knowledge.

Finding all the optical and spin-phonon parameters relevant to our proposal for a single material has proven to be a daunting task since this specific term of spin-phonon coupling has not been studied much. Also, there is little overlap between such magnetoelastic and IR studies. Hence, in our considerations below, we choose typical parameter values for different materials that have been measured in experiments. We hope that our theoretical proposal will motivate first-principles calculations and/or experiments to characterize the relevant noncentrosymmetric magnets with a focus on this proposal.

1.6 Estimating the spin-phonon coupling strength

As discussed and motivated above, Eq. (S3) for acoustic phonons can be employed for their $q = 0$ optical counterpart since the latter can be visualized as $q = \pi/a$ acoustic phonons in an extended Brillouin zone scheme, where a is the lattice constant. Below, we consider the optically-active longitudinal phonon mode polarized along the z direction. The differently polarized

phonons can be considered in an analogous manner. Further, we disregard any transverse phonon modes here.

As per Ref. [17], the effective longitudinal strain can be related with the canonical position $w(\mathbf{r})$ by $u_{zz}(\mathbf{r}) = 2w(\mathbf{r})/(a\rho)$, where ρ is the material density. Quantizing the system as detailed in Ref. [18], we directly obtain

$$u_{zz}(\mathbf{r}) = \frac{1}{a}\int \frac{\mathrm{d}\mathbf{q}}{(2\pi)^3}\sqrt{\frac{2\hbar}{\rho\Omega(\mathbf{q})}}\left[\beta(\mathbf{q})e^{i\mathbf{q}\cdot\mathbf{r}} + \beta^\dagger(\mathbf{q})e^{-i\mathbf{q}\cdot\mathbf{r}}\right] , \tag{S23}$$

where $\Omega(\mathbf{q})$ is the phonon frequency. The operators $\beta(\mathbf{q})$ and $\beta^\dagger(\mathbf{q})$ pertain to a continuum description. Within a small nanoparticle, only a uniform mode is optically active. We thus express the quantities in terms of discrete $\mathbf{q}$ values and keep only the $\mathbf{q} = 0$ mode, obtaining

$$u_{zz} = \frac{1}{a}\sqrt{\frac{2\hbar}{\rho V\Omega}}\left(\beta + \beta^\dagger\right), \tag{S24}$$

where V is the volume of the nanoparticle. Therefore, the interaction between the spin and the phonon operators with the help of Eqs. (S3) and (S24), reads

$$H_{S-P} = b\frac{S_z^2}{S^2}\left(\beta + \beta^\dagger\right) , \tag{S25}$$

where $b = b_1\sqrt{2\hbar V/(\rho\Omega a^2)}$ quantifies the spin-phonon interaction and has units of energy.

2 Supplementary Note 2: Polaritonic coupling

Equation (1) of the main text can be written in the form $H = H_{\mathrm{S}} + H_{\mathrm{S-P}} + H_{\mathrm{PP}}$ with

$$H_{\mathrm{PP}} = H_{\mathrm{P}} + H_{\mathrm{EM}} + H_{\mathrm{EM-P}} = \begin{pmatrix}\boldsymbol{\beta}^\dagger & \boldsymbol{\alpha}^\dagger\end{pmatrix}\mathbf{H}_{\mathrm{PP}}\begin{pmatrix}\boldsymbol{\beta}\\ \boldsymbol{\alpha}\end{pmatrix}. \tag{S26}$$

with $\mathbf{H}_{\mathrm{PP}}$ as defined in Eq. (8) of the main text. Considering $\mathbf{U} = \begin{pmatrix}\mathbf{C}\\ \mathbf{A}\end{pmatrix}$ to be the unitary matrix that diagonalizes $\mathbf{H}_{\mathrm{PP}}$, $\mathbf{U}^\dagger\mathbf{H}_{\mathrm{PP}}\mathbf{U} = \tilde{\boldsymbol{\omega}}$, and defining $\boldsymbol{\pi} = \mathbf{U}^\dagger\begin{pmatrix}\boldsymbol{\beta}\\ \boldsymbol{\alpha}\end{pmatrix}$, H_{PP} can be rewritten as

$$H_{\mathrm{PP}} = \sum_m \hbar\tilde{\omega}_m\pi_m^\dagger\pi_m , \tag{S27}$$

with π_m, $\pi_m^\dagger$ the creation and annihilation operators of the m-th polaritonic mode. Then, the total Hamiltonian can be written in the form

$$H = H_{\mathrm{S}} + \sum_m \hbar\tilde{\omega}_m \pi_m^\dagger \pi_m + \sum_{j,m,k} b_k \frac{S_{j;\,k}^2}{S^2}\left[(C_{jm}^k)^* \pi_m + C_{jm}^k \pi_m^\dagger\right] , \tag{S28}$$

where C_{jm}^k relate the polaritonic operators to the phononic ones.

With the help of Supplementary Note 3, the polaritonic operators can be integrated out and an effective spin-spin interaction is obtained from Eq. (S28), quantified by the coupling strength

$$\tilde{\mathbf{\Lambda}}_{j,j'} = \mathrm{Re}\left(b^2 \mathbf{C}_j \tilde{\boldsymbol{\omega}}^{-1} \mathbf{C}_{j'}^\dagger\right) . \tag{S29}$$

Now, we can express the spin-spin coupling through the inverse of $\mathbf{H}_{\mathrm{PP}}$. Using the explicit form of the matrix $\mathbf{H}_{\mathrm{PP}}$

$$\mathbf{H}_{\mathrm{PP}} = \begin{pmatrix} \mathbf{\Omega} & \mathbf{g} \\ \mathbf{g}^\dagger & \boldsymbol{\omega} \end{pmatrix} \tag{S30}$$

its inverse can be written as

$$\mathbf{H}_{\mathrm{PP}}^{-1} = \begin{pmatrix} (\mathbf{\Omega} - \mathbf{g}\boldsymbol{\omega}^{-1}\mathbf{g}^\dagger)^{-1} & -(\mathbf{\Omega} - \mathbf{g}\boldsymbol{\omega}^{-1}\mathbf{g}^\dagger)^{-1}\mathbf{g}\boldsymbol{\omega}^{-1} \\ -(\boldsymbol{\omega} - \mathbf{g}^\dagger\mathbf{\Omega}^{-1}\mathbf{g})^{-1}\mathbf{g}^\dagger\mathbf{\Omega}^{-1} & (\boldsymbol{\omega} - \mathbf{g}^\dagger\mathbf{\Omega}^{-1}\mathbf{g})^{-1} \end{pmatrix} . \tag{S31}$$

Since $\mathbf{U}\tilde{\boldsymbol{\omega}}^{-1}\mathbf{U}^\dagger = \mathbf{H}_{\mathrm{PP}}^{-1}$ and $\mathbf{U} = (\mathbf{C}\ \mathbf{A})^{\mathrm{T}}$, we obtain with the help of Eq. (S31)

$$\tilde{\mathbf{\Lambda}}_{j,j'} = \mathrm{Re}\left\{b^2\left[(\mathbf{\Omega} - \boldsymbol{\xi})^{-1}\right]_{jj'}\right\} , \tag{S32}$$

where

$$\boldsymbol{\xi} = \mathbf{g}\boldsymbol{\omega}^{-1}\mathbf{g}^\dagger = \begin{pmatrix} \boldsymbol{\xi}_{11} & \boldsymbol{\xi}_{12} & \cdots & \boldsymbol{\xi}_{1N_p} \\ \boldsymbol{\xi}_{21} & \boldsymbol{\xi}_{22} & \cdots & \boldsymbol{\xi}_{2N_p} \\ \vdots & \vdots & \vdots & \vdots \\ \boldsymbol{\xi}_{N_p1} & \boldsymbol{\xi}_{N_p2} & \cdots & \boldsymbol{\xi}_{N_pN_p} \end{pmatrix} , \tag{S33}$$

with N_p indicating the total number of nanoparticles and

$$\boldsymbol{\xi}_{jj'} = d_j d_{j'} \sum_{n=1}^{N_{\mathrm{cav}}} \frac{\mathbf{E}_n(\mathbf{r}_j) \otimes \mathbf{E}_n^*(\mathbf{r}_{j'})}{\hbar\omega_n} . \tag{S34}$$

$\otimes$ denotes the dyadic product between two vectors and N_{cav} indicates the total number of electromagnetic modes.

We note that $\mathbf{\Omega}$ has dimensions $3N_p \times 3N_p$, $\mathbf{g}$ has dimensions $3N_p \times M$, $\boldsymbol{\omega}$ has dimensions $M \times M$, and $\tilde{\mathbf{\Lambda}}_{j,j'}$ has dimensions 3×3, where $M = N_{\mathrm{cav}} + 3N_p$.

3 Supplementary Note 3: Effective spin-spin Hamiltonian

An effective spin-spin coupling is derived, based on the spin-phonon-photon interaction, by tracing out the polaritonic degrees of freedom. To do so, we employ the theoretical framework detailed in Refs. [19–23], which is based on the Euclidean path integral formulation.

The thermodynamic functions can be calculated from the canonical partition function $Z = \mathrm{Tr}[\exp(-\beta H)]$ with $\beta = 1/(k_B T)$, H the total Hamiltonian of the system and k_B the Boltzmann constant. A convenient basis to calculate the trace of the partition function of the bosonic polaritonic modes is formed by the coherent states $|\alpha\rangle$, which are eigenstates of the annihilation operator a, i.e., $a|\alpha\rangle = \alpha|\alpha\rangle$, and form a complete set, $\frac{1}{\pi}\int \mathrm{d}^2\alpha|\alpha\rangle\langle\alpha| = 1$, where the integral is over real and imaginary parts, $\int \mathrm{d}^2\alpha = \iint \mathrm{dRe}[\alpha]\,\mathrm{dIm}[\alpha]$.

Considering the total Hamiltonian to be of the form

$$H = H_\mathrm{S} + \sum_i \hbar\omega_i a_i^\dagger a_i + \sum_{i,j} \lambda S_j^2(\gamma_{ji} a_i + \gamma_{ji}^* a_i^\dagger) \ . \tag{S35}$$

and assuming that there are N spins and M polaritonic modes we have

$$Z = \frac{1}{\pi^M} \sum_{s_1} \cdots \sum_{s_N} \int \mathrm{d}^2\alpha_1 \ldots \int \mathrm{d}^2\alpha_M \\ \times \langle s_1 \ldots s_N | \langle \alpha_1 \ldots \alpha_M | \exp(-\beta H) | \alpha_1 \ldots \alpha_M \rangle | s_1 \ldots s_N \rangle \ , \tag{S36}$$

where the sum is taken over all spin states. By tracing out the polaritonic degrees of freedom, we wish to obtain an effective Hamiltonian such that

$$Z = \sum_{s_1} \cdots \sum_{s_N} \langle s_1 \ldots s_N | \exp(-\beta H_\mathrm{eff}) | s_1 \ldots s_N \rangle \ , \tag{S37}$$

with

$$\exp(-\beta H_\mathrm{eff}) = Z_\alpha = \frac{1}{\pi^M} \int \mathrm{d}^2\alpha_1 \ldots \int \mathrm{d}^2\alpha_M \langle \alpha_1 \ldots \alpha_M | \exp(-\beta H) | \alpha_1 \ldots \alpha_M \rangle \ . \tag{S38}$$

In order to accomplish this tracing out of the bosonic modes, we employ the result [20–23]

$$\langle \alpha_i | \exp(-\beta H) | \alpha_i \rangle \approx \exp\left\{ -\beta \left[H_\mathrm{S} + \hbar\omega_i \alpha_i^* \alpha_i + \sum_j \lambda S_j^2 (\gamma_{ji}\alpha_i + \gamma_{ji}^* \alpha_i^*) \right] \right\} \ . \tag{S39}$$

and pause to discuss it. Equation (S39) above is tantamount to replacing the operators a_i by their expectation values α_i in the coherent state. In this sense, it effectively disregards the quantum commutations between operators and is

reminiscent of an analogous replacement procedure within the path integral framework for integrating out excitations [22]. Strictly speaking, Eq. (S39) yields the exact result for the free energy per spin in the thermodynamic limit, i.e., when there are a large number of spins ($N \to \infty$). It has been justified semi-rigorously for a finite number M of the bosonic modes [19, 20]. However, since the number of spins is never infinity, one can consider and employ Eq. (S39) as an approximation that introduces a usually small error depending on N. In this spirit, Eq. (S39) has been successfully and widely employed for capturing the essential physics with a good enough accuracy [19–21, 23]. We also follow this procedure here. Since the modes are not interacting $Z_\alpha = \exp(-\beta H_\mathrm{S}) \prod_i Z_{\alpha_i}$ with

$$Z_{\alpha_i} = \frac{1}{\pi} \int \mathrm{d}^2\alpha_i \exp\Big\{ -\beta \Big[\hbar\omega_i \alpha_i^* \alpha_i + \sum_j \lambda S_j^2 (\gamma_{ji}\alpha_i + \gamma_{ji}^* \alpha_i^*) \Big] \Big\} . \tag{S40}$$

The integrals have Gaussian form and we get

$$Z_{\alpha_i} = \frac{1}{\beta\hbar\omega_i} \exp\Big[\beta \sum_{j,j'} \lambda S_j^2 \frac{\mathrm{Re}(\gamma_{ji}\gamma_{j'i}^*)}{\hbar\omega_i} \lambda S_{j'}^2 \Big] . \tag{S41}$$

Then, the total partition function Z_α takes the form

$$Z_\alpha = \exp(-\beta H_\mathrm{S}) \prod_i \Big\{ \frac{1}{\beta\hbar\omega_i} \exp\Big[\beta \sum_{j,j'} \lambda S_j^2 \frac{\mathrm{Re}(\gamma_{ji}\gamma_{j'i}^*)}{\hbar\omega_i} \lambda S_{j'}^2 \Big] \Big\} . \tag{S42}$$

In the thermodynamic limit [20, 21], we obtain the effective Hamiltonian

$$\begin{aligned} H_\mathrm{eff} &= H_\mathrm{S} - \sum_{j,j'} S_j^2 \tilde{\Lambda}_{j,j'} S_{j'}^2 \\ \tilde{\Lambda}_{j,j'} &= \mathrm{Re}[\lambda^2 \boldsymbol{\gamma}_j \boldsymbol{\omega}^{-1} \boldsymbol{\gamma}_{j'}^\dagger] , \end{aligned} \tag{S43}$$

where $\boldsymbol{\omega} = \mathrm{diag}(\hbar\omega_1, \hbar\omega_2, \ldots, \hbar\omega_M)$, and $\boldsymbol{\gamma}$ is a vector of length M with elements γ_{ji}. We note that the generalization for a spin operator with three components is straightforward, with the coupling $\tilde{\Lambda}$ becoming a 3×3 tensor.

4 Supplementary Note 4: Polaritonic coupling for continuum of EM modes

In the derivation above, a discrete number of modes has been considered. Here, we generalize the analysis for a continuum of electromagnetic modes.

We follow a macroscopic quantum electrodynamic treatment [24, 25], in which the quantized electric field is expressed as

$$\mathbf{E}(\mathbf{r}) = \sum_{\lambda} \int_0^{\infty} \mathrm{d}\omega \int \mathrm{d}^3 r' \tilde{\mathbf{G}}_{\lambda}(\mathbf{r}, \mathbf{r}', \omega) \mathbf{f}_{\lambda}(\mathbf{r}', \omega) + \text{H.c.} \;, \tag{S44}$$

with $\mathbf{f}_{\lambda}(\mathbf{r}', \omega)$ the bosonic annihilation operators of the EM modes, λ an index labeling the electric and magnetic contributions and $\tilde{\mathbf{G}}_{\lambda}(\mathbf{r}, \mathbf{r}', \omega)$ functions related to the (classical) dyadic Green's function of Maxwell's equations. The electromagnetic interaction of the EM fields with the nanoparticle dipoles in Coulomb gauge within the Power-Zienau-Woolley picture [26] and using the long-wavelength approximation is

$$H_{\mathrm{EM-P}} = \sum_{j} \sum_{\lambda} \int_0^{\infty} \mathrm{d}\omega \int \mathrm{d}^3 r' \mathbf{d}_j \cdot [\tilde{\mathbf{G}}_{\lambda}(\mathbf{r}, \mathbf{r}', \omega) \mathbf{f}_{\lambda}(\mathbf{r}', \omega)] \beta_j + \text{H.c.} \;. \tag{S45}$$

By formally discretizing the continuum of cavity modes and expressing it through a collective index $n = \{\lambda, l, \mathbf{r}', \omega\}$ (where l denotes Cartesian components), Eq. (S34) can be expressed in terms of the Green's functions

$$\begin{aligned} \xi_{j,j'}^{kk'} &= \sum_{n} \frac{g_{jn}^{k} g_{nj'}^{k'*}}{\hbar \omega_n} = d_i d_j \sum_{i} \frac{1}{\hbar \omega_i} \sum_{\lambda, l} \int \mathrm{d}^3 r' G_{\lambda}^{kl}(\mathbf{r}_j, \mathbf{r}', \omega_i) [G_{\lambda}^{k'l}(\mathbf{r}_{j'}, \mathbf{r}', \omega_i)]^{*\mathrm{T}} \\ &= d_j d_{j'} \sum_{i} \frac{\omega_i}{\pi \epsilon_0 c^2} \operatorname{Im} G^{kk'}(\mathbf{r}_j, \mathbf{r}_{j'}, \omega_i), \end{aligned} \tag{S46}$$

where we have used the property $\sum_{\lambda} \int \mathrm{d}^3 r' \tilde{\mathbf{G}}_{\lambda}(\mathbf{r}_j, \mathbf{r}', \omega) (\tilde{\mathbf{G}}_{\lambda}(\mathbf{r}_{j'}, \mathbf{r}', \omega))^{*\mathrm{T}} = \hbar \omega^2 \operatorname{Im} \tilde{\mathbf{G}}(\mathbf{r}_j, \mathbf{r}_{j'}, \omega)/(\pi \epsilon_0 c^2)$, where ϵ_0 is the vacuum electric permittivity and c the speed of light in vacuum. We note that we assumed that each particle has three equivalent dipole (phonon) directions with the same dipole moment d_j. After taking the continuum limit, we obtain

$$\tilde{\boldsymbol{\xi}}_{j,j'} = \frac{d_j d_{j'}}{\pi \epsilon_0 c^2} \int_0^{\infty} \mathrm{d}\omega \omega \operatorname{Im} \tilde{\mathbf{G}}(\mathbf{r}_j, \mathbf{r}_{j'}, \omega) \;. \tag{S47}$$

To analytically evaluate the integral, we use $\operatorname{Im} z = \frac{z - z^*}{2i}$ together with the property $\tilde{\mathbf{G}}^*(\mathbf{r}_i, \mathbf{r}_j, \omega) = \tilde{\mathbf{G}}(\mathbf{r}_i, \mathbf{r}_j, -\omega)$, giving

$$\int_0^{\infty} \mathrm{d}\omega \omega \operatorname{Im} \tilde{\mathbf{G}}(\mathbf{r}_i, \mathbf{r}_j, \omega) = \frac{1}{2i} \int_{-\infty}^{\infty} \mathrm{d}\omega \omega \tilde{\mathbf{G}}(\mathbf{r}_i, \mathbf{r}_j, \omega) \;. \tag{S48}$$

The function $\omega \tilde{\mathbf{G}}(\mathbf{r}_i, \mathbf{r}_j, \omega)$ has a simple pole at $\omega = 0$, and no other poles on the real axis or upper complex half space [24, 27]. Contour integration then yields the residue at $\omega = 0$, i.e., $\int_{-\infty}^{\infty} \mathrm{d}\omega \omega \tilde{\mathbf{G}}(\mathbf{r}_i, \mathbf{r}_j, \omega) = i\pi \left[\omega^2 \tilde{\mathbf{G}}(\mathbf{r}_i, \mathbf{r}_j, \omega)\right]_{\omega=0}$,

such that the polaritonic coupling of Eq. (S47) reads

$$\tilde{\boldsymbol{\xi}}_{j,j'} = \frac{d_j d_{j'}}{2\epsilon_0 c^2} \left[\omega^2 \tilde{\mathbf{G}}(\mathbf{r}_j, \mathbf{r}_{j'}, \omega)\right]_{\omega=0} . \tag{S49}$$

We note that this expression diverges for $j = j'$ due to the singular nature of the free-space Green's function, i.e., due to the divergence of the dipole self-energy. However, this divergence is due to the fact that the long-wavelength approximation means that the nanoparticles behave as point dipoles, and a more careful evaluation would lead to a (small) finite value [24, 28]. Since these terms just induce a constant energy shift, we instead assume that their contribution is already included in the bare system parameters and discard them in the following. Consequently, for couplings smaller than the phonon energy and for $j \neq j'$, from Eq. (S32) we obtain

$$\tilde{\mathbf{\Lambda}}_{j,j'} = \frac{b^2 d_j d_{j'}}{2\epsilon_0 c^2 \hbar^2 \Omega^2} \,\mathrm{Re}\left[\omega^2 \tilde{\mathbf{G}}(\mathbf{r}_j, \mathbf{r}_{j'}, \omega)\right]_{\omega=0} . \tag{S50}$$

It is worth noting that before obtaining the analytical expression of the coupling, Eq. (S50), it has been expressed as an integral over all frequencies (see Eq. (S47)). This characteristic offers a link with the van den Waals materials. These are described by nonlocal correlation energy functionals, which are expressed in terms of a kernel and the electron density. The former can be expressed as a frequency integral over all plasmon frequencies and is based on the virtual charge-density fluctuations of the electron gas [29]. It can be interpreted that the electron and the associated exchange-correlation hole form an antenna of charged parts [30]. Similarly, in our case, it can be interpreted that the phonons act as antennas.

5 Supplementary Note 5: Parameters

For our calculations we consider typical values of magnetic materials. Specifically, the magnetoelastic constant of EuIG $b_1 = 10^6$ J/m^3 [31], density $\rho = 5.4$ g/cm^3 [32, 33] of YIG, typical lattice constant $a = 1.1$ nm [32, 33] of ferrites and radius $r = 100$ nm yielding $b = 0.094$ eV. As discussed above in Sec. 1, we are not aware of any material for which all the material parameters required to quantify our proposed effect have been measured. Hence, we have taken typical experimental values available in the literature.

Also, we assume a dielectric function given by the Drude-Lorentz model with a single oscillator at angular frequency Ω, oscillator strength f_p, loss factor f_γ, and background permittivity ϵ_{bg}

$$\epsilon = \epsilon_{bg} + \frac{f_p \Omega^2}{\Omega^2 - \omega^2 - i f_\gamma \Omega \omega} . \tag{S51}$$

By comparing the polarizability of a spherical particle in the quasistatic approximations with that of a system with background polarizability and a single dipole transition with dipole moment d, we obtain

$$d = \frac{1}{\epsilon_{bg}+2}\sqrt{\frac{9\epsilon_0 \hbar V f_p \Omega}{2f_0}}$$
$$f_0 = \sqrt{1 + \frac{f_p}{(\epsilon_{bg}+2)} - \frac{f_\gamma}{4}} \,. \tag{S52}$$

For $\epsilon_{bg} = 1$, $f_p = 1$, $f_\gamma = 0.01$, and $\Omega/(2\pi) = 100$ cm^{-1}, which correspond to typical values of iron garnets and cuprates [34–36], the dipole moment of each nanoparticle equals about 1.7 kD.

We note that our material parameters are within realistic range and in order to reach convergence, an 1600×1600-nanoparticle array is needed.

6 Supplementary Note 6: Mean-field framework

According to the mean field approach, each spin fluctuates around its mean value such that we may expand the spin operator as $\mathbf{S} = \langle \mathbf{S} \rangle + \delta \mathbf{s}$, with $\delta s \ll \langle S \rangle$. With this approach, for the k-th component we have $(S_{i;\,k})^2 = \left(\langle S_{i;\,k}\rangle + \delta s_{i;\,k}\right)^2 \approx \langle S_{i;\,k}\rangle^2 + 2\langle S_{i;\,k}\rangle \delta s_{i;\,k}$ keeping up to first-order terms in $\delta s_{i;\,k}$. Thus, a single term of Eq. (4) in the main text takes the form

$$(S_{j;\,k})^2 \tilde{\Lambda}^{kk'}_{j,j'} (S_{j';\,k'})^2 \approx -3\langle S_{j;\,k}\rangle^2 \tilde{\Lambda}^{kk'}_{j,j'} \langle S_{j';\,k'}\rangle^2 + 2\langle S_{j;\,k}\rangle^2 \tilde{\Lambda}^{kk'}_{j,j'} \langle S_{j';\,k'}\rangle S_{j;\,k'}$$
$$+ 2S_{j;\,k}\langle S_{j;\,k}\rangle \tilde{\Lambda}^{kk'}_{j,j'} \langle S_{j';\,k'}\rangle^2 \,, \tag{S53}$$

where we have further employed $\delta s_{i;\,k} = S_{i;\,k} - \langle S_{i;\,k}\rangle$. Assuming that the mean value of all spins is the same, Eq. (4) of the main text can be cast in the form

$$H_{\mathrm{MF}} = \frac{3}{S^4}\langle \mathbf{S}\rangle^2 \cdot \mathbf{\Lambda} \cdot \langle \mathbf{S}\rangle^2 - \frac{1}{S}\sum_j \mathbf{h}_j \cdot \mathbf{S}_j \,, \tag{S54}$$

where $\langle \mathbf{S}\rangle^2 \equiv (\langle S_x\rangle^2, \langle S_y\rangle^2, \langle S_y\rangle^2)$, $\mathbf{\Lambda} = \sum_{j,j'} \tilde{\mathbf{\Lambda}}_{j,j'}$, $\mathbf{h}_j = 2\boldsymbol{\sigma}\left(\mathbf{\Lambda}_j + \mathbf{\Lambda}_j^{\mathrm{T}}\right)\langle \mathbf{S}\rangle / S^3$, $\mathbf{\Lambda}_j = \sum_{j'} \tilde{\mathbf{\Lambda}}_{j,j'}$, and $\boldsymbol{\sigma} = \mathrm{diag}(\langle S_x\rangle^2, \langle S_y\rangle^2, \langle S_y\rangle^2)$. We note that H_{S} has been neglected, which can be realized by a Zeeman term for switched off external magnetic field. It can be shown that for the hexagonal/square arrays in the x-y plane, the mean-field coupling tensor is diagonal and then

$$\mathbf{h}_j = \frac{4}{S^3}\left(\Lambda_{j;\,x}\langle S_x\rangle^3 \widehat{\mathbf{x}} + \Lambda_{j;\,y}\langle S_y\rangle^3 \widehat{\mathbf{y}} + \Lambda_{j;\,z}\langle S_z\rangle^3 \widehat{\mathbf{z}}\right) \,, \tag{S55}$$

$\Lambda_{k;\,j} = \sum_i \tilde{\Lambda}^{kk}_{j,j'}$ for $k = x, y, z$.

Interestingly, the mean-field Hamiltonian, Eq. (S54), has the same form as in the case of conventional ferromagnetism, in which $\mathbf{h}_j$ can be viewed as an, effective, internal molecular magnetic field. Following the standard procedure of the mean-field theory [32, 37], the self-consistent equation for the expectation value of the k-th component of the spin operator for the j-th nanoparticle is

$$\langle S_k \rangle = \frac{h_k}{h} S B_S(\beta h) \ , \tag{S56}$$

where $B_S(x) = \frac{2S+1}{2S} \coth \left[\frac{(2S+1)x}{2S}\right] - \frac{1}{2S} \coth \left(\frac{x}{2S}\right)$ is the Brillouin function and $h = 4\sqrt{\sum_k (\Lambda_k \langle S_k \rangle^3)^2}$. We note that the j dependence has been dropped because all spins are identical.

In the limit of $S \to \infty$ the Brillouin function reduces to the Langevin function, $L(x) = -1/x + \coth(x)$, and the self-consistent equation reads

$$\frac{\langle S_x \rangle}{S} = \frac{h_k}{h} \left[-\frac{1}{\beta h} + \coth(\beta h) \right] \ . \tag{S57}$$

The Helmholtz free energy, $F = U - TS$, reads

$$F = \frac{N}{\beta} \left\{ \frac{3\beta}{S^4} \sum_k (\Lambda_k^2 \langle S_k \rangle^4) - \ln \left[\cosh(\beta h) + \coth\left(\frac{\beta h}{2S}\right) \sinh(\beta h) \right] \right\} \ . \tag{S58}$$

7 Supplementary Note 7: Validating the long-wavelength approximation

The effective spin-spin coupling [Eq. (S50)] is obtained by carrying out a contour integration, with the contour including the entire real frequency axis [see Eqs. (S47) - (S50)]. In practice, the long-wavelength or dipole approximation employed in our treatment of the photon-phonon coupling is valid up to frequencies with the corresponding optical wavelength much larger than the nanoparticle size. This imposes a physical restriction on the quantity that is considered ∞ in the frequency integral Eq. (S47). Here, we demonstrate that imposing the appropriate physical frequency cut-off, instead of carrying out the integral to ∞, still yields the same result for the physical systems considered herein. Such considerations and results are common in various physical phenomena when a formally infinite value needs to be replaced by a high cut-off governed by certain physical constraints.

We consider the free-space dyadic Green's function

$$\tilde{\mathbf{G}}_0(\mathbf{r}_i, \mathbf{r}_j, \omega) = \frac{e^{ikr}}{4\pi r} \left[\tilde{\mathbf{I}} - \widehat{\mathbf{r}} \otimes \widehat{\mathbf{r}} + \frac{(ikr-1)}{k^2 r^2} (\tilde{\mathbf{I}} - 3\widehat{\mathbf{r}} \otimes \widehat{\mathbf{r}}) \right], \tag{S59}$$

where $k = \omega/c$ is the optical wavenumber, $\tilde{\mathbf{I}}$ is the 3×3 identity matrix, $\mathbf{r} = \mathbf{r}_i - \mathbf{r}_j$, and $\widehat{\mathbf{r}} = \mathbf{r}/r$.

We are interested in the integral

$$\tilde{\mathbf{I}}_0 = \frac{1}{\pi\epsilon_0 c^2} \int_0^\infty \mathrm{d}\omega\omega \,\mathrm{Im}\, \tilde{\mathbf{G}}_0(\mathbf{r}_i, \mathbf{r}_j, \omega) \;, \tag{S60}$$

which, using contour integration, can be calculated analytically (see Supplementary Note 4)

$$\tilde{\mathbf{I}}_0 = \frac{1}{2\epsilon_0 c^2} \left[\omega^2 \tilde{\mathbf{G}}_0(\mathbf{r}_i, \mathbf{r}_j, \omega)\right]_{\omega=0} \;. \tag{S61}$$

By inserting Eq. (S59) into Eq. (S61) yields the kernel of the free-space electrostatic dipole-dipole interaction energy as per the expectations

$$\tilde{\mathbf{I}}_0 = \frac{1}{2} \frac{3\widehat{\mathbf{r}} \otimes \widehat{\mathbf{r}} - \tilde{\mathbf{I}}}{4\pi\epsilon_0 r^3}, \tag{S62}$$

where the factor 1/2 accounts for each pair of dipole appearing twice in the sum over i and j.

We next evaluate the integral in Eq. (S60) with an exponential cutoff at wavelength λ_c, i.e., a cut-off frequency of $\omega_c = 2\pi c/\lambda_c$. To simplify the expressions, we multiply the denominator of Eq. (S62) by $8\pi\epsilon_0 r^3$, yielding

$$\begin{aligned} 8\pi\epsilon_0 r^3 \tilde{\mathbf{I}}_{\omega_c} &= \frac{8r^3}{c^2} \int_0^\infty \mathrm{d}\omega\omega \,\mathrm{Im}\, \mathbf{G}_0(\mathbf{r}_i, \mathbf{r}_j, \omega) \mathrm{e}^{-\omega^2/\omega_c^2} \\ &= 4\pi^{5/2} r_\lambda^3 e^{-\pi^2 r_\lambda^2} \left(\tilde{\mathbf{I}} - \widehat{\mathbf{r}} \otimes \widehat{\mathbf{r}}\right) + \\ &\quad \left(1 - \mathrm{erfc}(\pi r_\lambda) - 2\sqrt{\pi} r_\lambda e^{-\pi^2 r_\lambda^2}\right) \left(3\widehat{\mathbf{r}} \otimes \widehat{\mathbf{r}} - \tilde{\mathbf{I}}\right), \end{aligned} \tag{S63}$$

where we have introduced $r_\lambda = r/\lambda_c$ for simplicity. In the limit $\lambda_c \to 0$ (i.e., $\omega_c \to \infty$), this clearly recovers Eq. (S62). Interestingly, this convergence with the increasing cut-off frequency ω_c is very fast, as all terms apart from the one obtained in Eq. (S62) are suppressed by $\mathrm{e}^{-\pi^2 r_\lambda^2}$ (which is also the asymptotic behavior of erfc). For $r_\lambda = 1$, the relative error is on the level of 0.4%, and for $r_\lambda = 2$, it is already below 10^{-14}. This implies that for a given distance r between points, a cutoff wavelength similar to that distance is sufficient to obtain a fully converged result. Since the spatial extent of our considered nanoparticles can at most be of the order of their separation (and is typically significantly smaller), this implies that the dipole approximation works well. The formal extension of the frequency integral to an upper limit of infinity without going beyond the dipole approximation is thus well-justified. We note that while we have here explicitly demonstrated the case of free space, the same arguments are expected to apply in general environments.

References

[1] Kittel, C.: Physical theory of ferromagnetic domains. Rev. Mod. Phys. **21**, 541–583 (1949). https://doi.org/10.1103/RevModPhys.21.541

[2] Chikazumi, S.: Physics of Ferromagnetism. International Series of Monographs on Physics. OUP Oxford, ??? (2009). https://books.google.es/books?id=AZVfuxXF2GsC

[3] Kamra, A., Keshtgar, H., Yan, P., Bauer, G.E.W.: Coherent elastic excitation of spin waves. Phys. Rev. B **91**, 104409 (2015). https://doi.org/10.1103/PhysRevB.91.104409

[4] Néel, Louis: Anisotropie magnétique superficielle et surstructures d'orientation. J. Phys. Radium **15**(4), 225–239 (1954). https://doi.org/10.1051/jphysrad:01954001504022500

[5] Mankovsky, S., Polesya, S., Lange, H., Weißenhofer, M., Nowak, U., Ebert, H.: Angular momentum transfer via relativistic spin-lattice coupling from first principles. Phys. Rev. Lett. **129**, 067202 (2022). https://doi.org/10.1103/PhysRevLett.129.067202

[6] Thingstad, E., Kamra, A., Brataas, A., Sudbø, A.: Chiral phonon transport induced by topological magnons. Phys. Rev. Lett. **122**, 107201 (2019). https://doi.org/10.1103/PhysRevLett.122.107201

[7] Go, G., Kim, S.K., Lee, K.-J.: Topological magnon-phonon hybrid excitations in two-dimensional ferromagnets with tunable chern numbers. Phys. Rev. Lett. **123**, 237207 (2019). https://doi.org/10.1103/PhysRevLett.123.237207

[8] Weiler, M., Huebl, H., Goerg, F.S., Czeschka, F.D., Gross, R., Goennenwein, S.T.B.: Spin pumping with coherent elastic waves. Phys. Rev. Lett. **108**, 176601 (2012). https://doi.org/10.1103/PhysRevLett.108.176601

[9] Brown, W.F.J.: Magnetoelastic Interactions. Springer Tracts in Natural Philosophy. Springer, ??? (1966). https://books.google.es/books?id=-5LAq-Po52wC

[10] Kikkawa, T., Shen, K., Flebus, B., Duine, R.A., Uchida, K.-i., Qiu, Z., Bauer, G.E.W., Saitoh, E.: Magnon polarons in the spin seebeck effect. Phys. Rev. Lett. **117**, 207203 (2016). https://doi.org/10.1103/PhysRevLett.117.207203

[11] Ashcroft, N.W., Mermin, N.D.: Solid State Physics. HRW international editions. Holt, Rinehart and Winston, ??? (1976). https://books.google.es/books?id=1C9HAQAAIAAJ

[12] Anand, L., Gurtin, M.E., Lele, S.P., Gething, C.: A one-dimensional theory of strain-gradient plasticity: Formulation, analysis, numerical results. Journal of the Mechanics and Physics of Solids **53**(8), 1789–1826 (2005). https://doi.org/10.1016/j.jmps.2005.03.003

[13] Curtis, J.B., Grankin, A., Poniatowski, N.R., Galitski, V.M., Narang, P., Demler, E.: Cavity magnon-polaritons in cuprate parent compounds. Phys. Rev. Res. **4**, 013101 (2022). https://doi.org/10.1103/PhysRevResearch.4.013101

[14] Garst, M., Waizner, J., Grundler, D.: Collective spin excitations of helices and magnetic skyrmions: review and perspectives of magnonics in non-centrosymmetric magnets. Journal of Physics D: Applied Physics **50**(29), 293002 (2017). https://doi.org/10.1088/1361-6463/aa7573

[15] Kanazawa, N., Seki, S., Tokura, Y.: Ferromagnetic materials: Non-centrosymmetric magnets hosting magnetic skyrmions (adv. mater. 25/2017). Advanced Materials **29**(25) (2017). https://doi.org/10.1002/adma.201770180

[16] Nomura, T., Zhang, X.-X., Zherlitsyn, S., Wosnitza, J., Tokura, Y., Nagaosa, N., Seki, S.: Phonon magnetochiral effect. Phys. Rev. Lett. **122**, 145901 (2019). https://doi.org/10.1103/PhysRevLett.122.145901

[17] Kittel, C., Fong, C.: Quantum Theory of Solids. John Wiley & Sons, Toronto (1987)

[18] Feynman, R.P.: Statistical Mechanics: A Set Of Lectures. CRC Press, Florida (1998). https://doi.org/10.1201/9780429493034

[19] Hepp, K., Lieb, E.H.: On the superradiant phase transition for molecules in a quantized radiation field: the dicke maser model. Annals of Physics **76**, 360 (1973)

[20] Wang, Y.K., Hioe, F.T.: Phase Transition in the Dicke Model of Superradiance. Physical Review A **7**(3), 831–836 (1973). https://doi.org/10.1103/PhysRevA.7.831

[21] Román-Roche, J., Luis, F., Zueco, D.: Photon condensation and enhanced magnetism in cavity qed. Phys. Rev. Lett. **127**, 167201 (2021). https://doi.org/10.1103/PhysRevLett.127.167201

[22] Coleman, P.: Introduction to Many-Body Physics. Cambridge University Press, Cambridge (2015). https://doi.org/10.1017/CBO9781139020916. https://www.cambridge.org/core/books/introduction-to-manybody-physics/B7598FC1FCEE0285F5EC767E835854C8

[23] Román-Roche, J., Zueco, D.: Effective theory for matter in non-perturbative cavity QED. SciPost Physics Lecture Notes, 50 (2022). https://doi.org/10.21468/SciPostPhysLectNotes.50. Accessed 2023-05-07

[24] Buhmann, S.Y.: Dispersion Forces I. Springer Tracts in Modern Physics, vol. 247. Springer, Berlin, Heidelberg (2012). https://doi.org/10.1007/978-3-642-32484-0

[25] Feist, J., Fernández-Domínguez, A.I., García-Vidal, F.J.: Macroscopic qed for quantum nanophotonics: emitter-centered modes as a minimal basis for multiemitter problems. Nanophotonics **10**(1), 477–489 (2021). https://doi.org/10.1515/nanoph-2020-0451

[26] Andrews, D.L., Jones, G.A., Salam, A., Woolley, R.G.: Perspective: Quantum Hamiltonians for optical interactions. J. Chem. Phys. **148**(4), 040901 (2018). https://doi.org/10.1063/1.5018399

[27] Novotny, L., Hecht, B.: Principles of Nano-Optics, 2nd edn. Cambridge University Press, Cambridge (2012). https://doi.org/10.1017/CBO9780511794193

[28] Casimir, H.B.G., Polder, D.: The Influence of Retardation on the London-van der Waals Forces. Phys. Rev. **73**(4), 360 (1948). https://doi.org/10.1103/PhysRev.73.360

[29] Chakraborty, D., Berland, K., Thonhauser, T.: Next-Generation Nonlocal van der Waals Density Functional. Journal of Chemical Theory and Computation **16**(9), 5893–5911 (2020). https://doi.org/10.1021/acs.jctc.0c00471. Publisher: American Chemical Society. Accessed 2023-07-25

[30] Shukla, V., Jiao, Y., Lee, J.-H., Schröder, E., Neaton, J.B., Hyldgaard, P.: Accurate nonempirical range-separated hybrid van der waals density functional for complex molecular problems, solids, and surfaces. Phys. Rev. X **12**, 041003 (2022). https://doi.org/10.1103/PhysRevX.12.041003

[31] Comstock, R.L.: Magnetoelastic coupling constants of the ferrites and garnets. Proceedings of the IEEE **53**(10), 1508–1517 (1965). https://doi.org/10.1109/PROC.1965.4263. Conference Name: Proceedings of the IEEE

[32] Coey, J.M.D.: Magnetism and Magnetic Materials, 1st edn. Cambridge University Press, ??? (2001). https://doi.org/10.1017/CBO9780511845000. https://www.cambridge.org/core/product/identifier/9780511845000/type/book Accessed 2023-02-17

[33] Stancil, D.D., Prabhakar, A.: Spin Waves: Theory and Applications. Springer, New York (2009). https://doi.org/10.1007/978-0-387-77865-5.

https://www.springer.com/gp/book/9780387778648 Accessed 2021-02-11

[34] Grunberg, P., Koningstein, J.A., Uitert, L.G.V.: Optical Phonons in Iron Garnets. JOSA **61**(12), 1613–1617 (1971). https://doi.org/10.1364/JOSA.61.001613. Publisher: Optica Publishing Group. Accessed 2022-11-30

[35] Bernhard, C., Holden, T., Humlícek, J., Munzar, D., Golnik, A., Kläser, M., Wolf, T., Carr, L., Homes, C., Keimer, B., Cardona, M.: In-plane polarized collective modes in detwinned YBa2Cu3O6.95 observed by spectral ellipsometry. Solid State Communications **121**(2), 93–97 (2002). https://doi.org/10.1016/S0038-1098(01)00451-3. Accessed 2022-01-12

[36] Feneberg, M., Nixdorf, J., Neumann, M.D., Esser, N., Artús, L., Cuscó, R., Yamaguchi, T., Goldhahn, R.: Ordinary dielectric function of corundumlike $\alpha - Ga_2O_3$ from 40 meV to 20 eV. Physical Review Materials **2**(4), 044601 (2018). https://doi.org/10.1103/PhysRevMaterials.2.044601. Publisher: American Physical Society. Accessed 2022-12-08

[37] Gurevich, A.G., Melkov, G.A.: Magnetization Oscillations and Waves. CRC Press, Boca Raton (1996). https://books.google.es/books?id=YgQtSvFIvFQC
